# Sarcopenia and Cognitive Decline in Hospitalized Older Adults from a Prospective Study

**DOI:** 10.14336/AD.2024.1676

**Published:** 2026-02-25

**Authors:** Sapir Kon-Kfir, Tali Cukierman-Yaffe, Haim Krupkin, Ana Belkin, Gadi Shlomai, Jonathan Bleier, Shiri Weinstein, Liora Bruckmayer, Elad Prinz, Alon Kaplan, Michal Goldenberg Shraga, Dana Lev, Shahar Dekel, Noa Shalmon, Nurit Tsarfaty, Niv Reiss, Evelyne Bischof, Avshalom Leibowitz

**Affiliations:** ^1^Department of Internal Medicine D, Sheba Medical Center at Tel Hashomer, Tel Hashomer, Israel.; ^2^Faculty of Medicine, Tel Aviv University, Tel Aviv, Israel.; ^3^Epidemiology department, School of public health Faculty of Medicine, Herczeg institute on aging, Tel Aviv University, Tel Aviv, Israel.; ^4^Division of Endocrinology, Diabetes and Metabolism, Sheba Medical Center, Ramat Gan, Israel.; ^5^Arrow Program for medical research education, Sheba Medical Center, Ramat Gan, Israel.; ^6^Department of Genetics, Stanford University, Stanford, CA 94305, USA.; ^7^Clinical immunology, Angioadema and Allergy institute, Sheba Medical Center at Tel Hashomer, Tel Hashomer, Israel.; ^8^Sheba Longevity Center, Sheba Medical Center at Tel Hashomer, Tel Hashomer, Israel.

**Keywords:** grip strength, frailty, clinical stamina assessment, agility, aging

## Abstract

As populations age, sarcopenia increasingly impacts healthcare due to its associations with morbidity, mortality, and cognitive decline. *This study is a cross-sectional analysis of prospectively collected data from 140 older adults hospitalized in an internal medicine department.* Sarcopenia was measured by handgrip strength, and cognitive function by the Digit Symbol Substitution Test (DSST). Sarcopenic patients (n=78) had lower DSST scores (p=0.003) and Norton scores (p<0.001) compared to non-sarcopenic patients. Handgrip strength showed a significant positive correlation with DSST scores (R=0.26, p=0.0019), persisting after adjustments for age and sex (R=0.42, p=1.7e-07). This study underscores a significant association between sarcopenia and cognitive decline in hospitalized older adults, advocating for routine sarcopenia and cognitive assessments upon admission. These findings emphasize the importance of identifying at-risk patients early and developing targeted interventions. Future research should further explore underlying mechanisms and validate findings in broader cohorts.

## INTRODUCTION

With advances in medicine and technology, life expectancy is constantly on the rise. According to the United Nations' *World Population Prospects 2022: Summary of Results* (available at www.un.org/development/desa/pd/sites/www.un.org.development.desa.pd/fil/es/wpp2022_summary_of_results.pdf, accessed 18/2/2025), in 2022, people over the age of 65 accounted for 10 percent of the global population, and this figure is expected to grow to 16 percent by 2050. Age-related processes are the main reason for morbidity, with older adults being at increased risk of developing chronic diseases and disabilities, which in turn also lead to hospitalizations [1, 2]. While chronic management in the outpatient setting provides more time and resources for diagnostic evaluations and therapeutic optimization, the acute hospitalization of elderly, comorbid patients necessitate a distinct set of skills and rapid diagnostic tools to enable swift prognostic and predictive differentiation. Given the rising prevalence of age-related conditions, simple, rapid screening methods are crucial for early identification of sarcopenia and cognitive decline in older adults

The official definition of sarcopenia is still under debate, but it can be often best referred to as a term describing criteria changes in body composition and function associated with age [3]. One of the most widely used definitions today is that of the European Working Group On Sarcopenia In Older People (EWGSOP2) which defines low muscle strength as the hallmark of sarcopenia, while muscle quantity or quality as well as physical performance are additional criteria for confirmation [4].The EWGSOP recommends using the grip strength and chair rise measurement as screening measurements [4]. Especially hand-grip strength is an inexpensive and simple method with a potential in the inpatient setting, allowing for a prognostic asset for mortality, disability, complications, and prolonged stay [5, 6]. Sarcopenia has a major impact on older adults, where sarcopenic patients are at greater risk for frailty, with events of falls, fractures, general functional decline and premature mortality [7-10].

In addition, patients with sarcopenia experience longer hospitalizations and have an increased risk of readmission leading to loss of independence, reduced quality of life and institutionalization [12], ultimately causing greater healthcare costs for patients, their caregivers and society at large [13]. The reported prevalence of sarcopenia varies greatly among studies, depending on cut-offs and classification criteria used. In a recent meta-analysis, the prevalence of sarcopenia in individuals ≥ 60 years old is 10 to 27 percent [14].

Dementia and mild cognitive impairment are independent risk factors for morbidity and mortality [15-17]. Several studies have demonstrated a link between sarcopenia and a higher risk of cognitive impairment [18-20]. Other data suggest that sarcopenia may worsen individual cognitive decline [21].

Skeletal muscle acts as an endocrine organ, secreting myokines that support neurogenesis and synaptic plasticity, both crucial for learning and memory function [22]. Some myokines also have anti-inflammatory effects, and their expression is modulated by physical activity, making inactivity a potential contributor to cognitive decline through downregulation of these neuroprotective factors [23]. Additionally, reduced muscle mass may lower brain-derived neurotrophic factor (BDNF), a key neurotrophin involved in neurogenesis and synaptic plasticity. Low BDNF levels are linked to Alzheimer’s disease, schizophrenia, depression, and neuro-degenerative disorders like Huntington’s and Parkinson’s [24]. Shared pathophysiological pathways, including oxidative stress, chronic inflammation, and insulin resistance, further connect sarcopenia and cognitive decline [25].

A bidirectional association between cognitive decline and sarcopenia has been described before in longitudinal real-world data, however no studies have been conducted in acute phase hospitalized patients.

As of now, the assessment for sarcopenia or cognitive status are not included in the standard vital at the admission even though both evaluations are vital for a comprehensive understanding of the patient’s status upon admission. One of the main reasons is a lack of validation of a concise assessment panel in the inpatient setting. The tools and techniques employed need to be not only reliable and robust but also efficient enough to justify the allocation of limited physician time. Thus, we used the index of muscle strength (hand grip strength) and a recently validated digital version of the Digit Symbol Substitution Test (DSST) [26]. The DSST requires individuals to match symbols to numbers within a limited timeframe, measuring both speed and accuracy. It is a straightforward format, language independent, and culturally neutral make it a widely utilized tool in clinical assessments. It demonstrated an ability to detect impairments across various conditions, including major depressive disorder and cognitive changes over time [27]. A study with 100 older adults with type 2 diabetes demonstrated a strong correlation (r = 0.76, p < 0.001) between the digital and traditional pencil-and-paper versions, indicating the digital DSST is a reliable tool for assessing cognitive function in this population, though further validation in self-administration and clinical settings is needed [26]. The digital DSST was developed by a multidisciplinary team focusing on user-friendliness and ease of use. The interface accurately captures user inputs, and the machine-learning model trained on hundreds of symbols from healthy volunteers achieved an overall accuracy of 89.1% during validation.

As far as we know, our study represents the first prospective study conducted in this specific population of hospitalized older adults, which is notably the most burdened by healthcare costs and permits limited time resources available for comprehensive screening measures. Given that this group carries the heaviest economic and logistical load on healthcare systems, the study's methodology must be rigorously justified and resonate with healthcare professionals. In sum, our reported research not only fills a critical gap in understanding but also serves as a foundational model for conducting further prospective designs and allow a viable method for studying causality between sarcopenia and cognitive status.

## MATERIALS AND METHODS

### Study Participants

This study is part of a broader investigation conducted among hospitalized patients within the internal medicine department of Sheba Medical Center, aiming to elucidate the correlation between sarcopenia and metabolic parameters. For this subpopulation analysis, 140 patients out of the 281 total participants, aged 65-95, who underwent cognitive assessment were included. Patients were excluded if they were unable to provide informed consent, had poor prognosis upon admission (such as advanced malignancy or a fatal condition with a life expectancy of less than three months), or were unable to perform cognitive and physical assessments. The cohort comprised both sarcopenic and control groups, formed based on handgrip strength criteria, to ensure methodological rigor.

### Measures

Handgrip Strength Assessment: Handgrip strength was assessed using a JAMAR dynamometer. Each participant completed the grip strength test three times with their dominant hand, and the average value was recorded.

Sarcopenia was defined using the EWGSOP2 criteria i.e. handgrip strength below 16 Kg for women and below 27 Kg for men.

**Figure 1.**
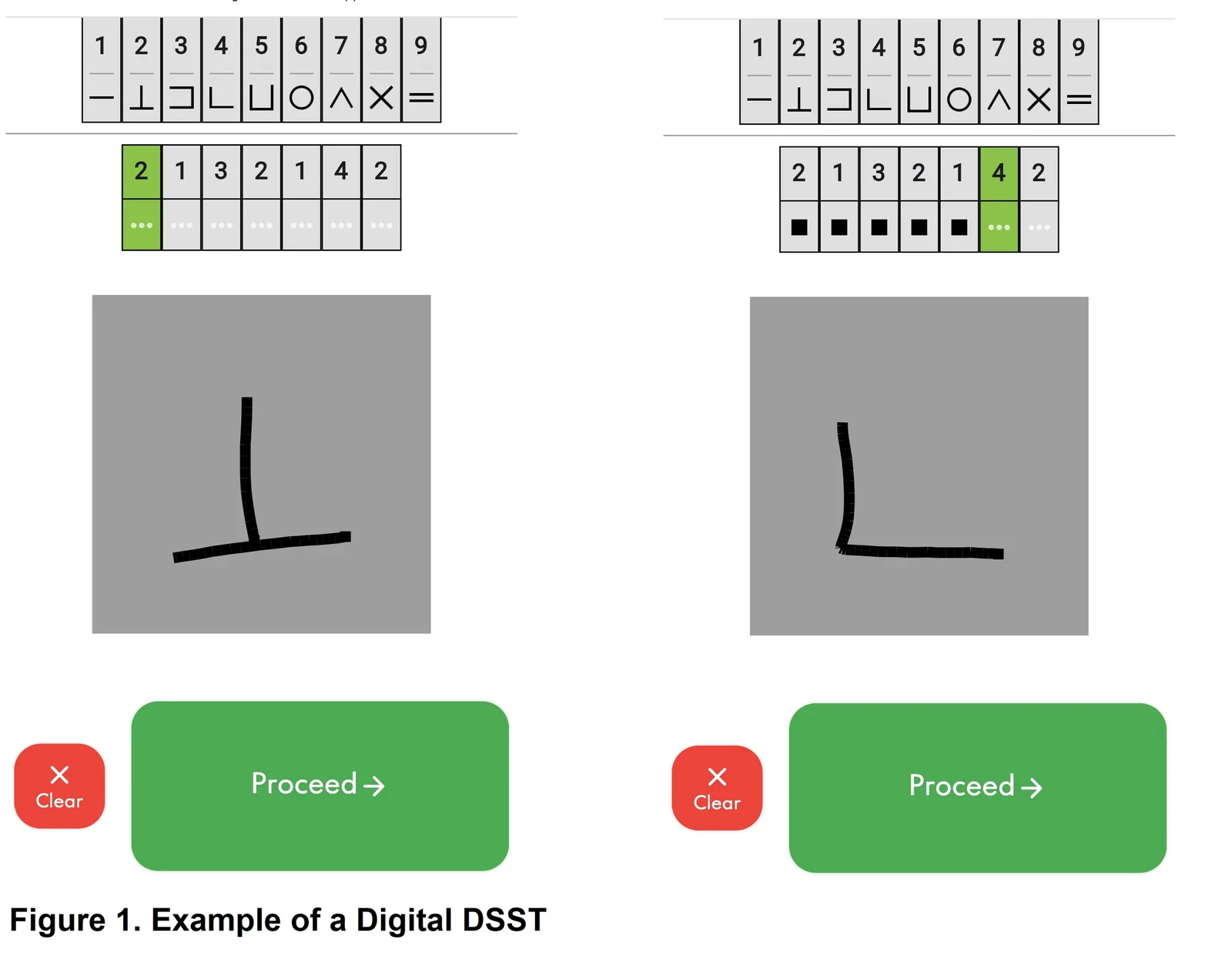
**Example of a Digital DSST**. Illustration of the Digit Symbol Substitution Test (DSST) used in cognitive assessment.

Digit Symbol Substitution Test (DSST): Participants completed a recently validated digital version of the DSST using a smartphone with a 6.52-inch screen (Fig. 1). After a brief explanation, patients were given a demonstration where they copied seven symbols according to an answer key. Following the demonstration, they were instructed to accurately copy as many symbols as possible within 120 seconds. Cognitive assessment using the DSST was performed for all patients in the morning, during their first 24 hours of admission. The decision to choose the digital DSST over other tests was based on its relative ease of administration, its non-language-specific nature, and the simplicity of using the digital version, which does not require special accessories.

Both tests (handgrip strength and DSST) were conducted by the same two-member team who adhered to a standardized protocol to ensure consistency.

### Data Collection

In addition to the handgrip strength and DSST results, relevant medical information was collected from the participants' medical records. This included details about their medical history, current diagnoses, vital signs, medications, and other relevant clinical information, including Norton score. Healthcare professionals use the Norton Scale in order to identify individuals at risk of developing pressure ulcers. The score comprises of five fields: physical condition, mental condition, activity, mobility, and incontinence. Each field is rated from one to four, with a maximum score of twenty. A lower score indicating a higher risk [28].

### Ethical Considerations

The study was conducted in accordance with the Declaration of Helsinki and approved by the institutional ethics committee. Informed consent was obtained from all participants before their inclusion in the study.

### Statistical analysis

For descriptive statistics we used mean, median, minimum, maximum, simple range, percent, and confidence interval. The difference in variables distribution was determined using a Chi-squared test, and a Mann-Whitney U test. All statistical analyses were conducted using R software, and significance levels were set at p-values <0.05. Linear regression was used to assess the relationship between hand grip strength and DSS score, before and after adjustment for age and sex.

We conducted additional analyses to explore baseline characteristics, including reasons for admission, total comorbidity burden using the Charlson Comorbidity Index (CCI), and medication profiles. To ensure patient anonymity, rare conditions and medications were excluded from the supplementary material.

**Table 1:** Patients’ characteristics.

Characteristic	Sarcopenic (n=78)	Non-Sarcopenic (n=62)	P.value
Median Age - Years	76.74 (75.12-78.36)	74.37 (72.68-76.06)	0.04
Female sex - no. (%)	43	51	0.46
Average hand grip pressure - Kg - Male	21.02 (19.81-22.23)	31.90 (30.39-33.42)	<0.001
Average hand grip pressure - Kg - Female	13.01 (11.93-14.09)	20.80 (19.66-21.94)	<0.001
Norton Score	18.33 (17.93-18.73)	19.16 (18.99-19.33)	0.001
BMI *(18-25)*	27.03 (25.26-28.8)	27.42 (26.17-28.68)	0.22
Diastolic Blood Pressure - mmHg	73.01 (70.52-75.5)	73.39 (71.06-75.72)	0.72
Systolic Blood Pressure - mmHg	139.76 (133.61-145.9)	135.42 (130.26-140.57)	0.39
Median hospitalization duration - Days	2 (1-23)	2 (1-8)	0.25
Albumin *(3.6-5.5 g/dl*)	3.68 (3.58-3.79)	3.85 (3.76-3.95)	0.01
Urea *(15-45 mg/dl)*	53.66 (46.37-60.94)	43.8 (37.92-49.69)	0.03
Creatinine *(0.51-0.95 mg/dl)*	1.21 (1.02-1.4)	0.97 (0.88-1.06)	0.19
Hemoglobin *(11.7-15.7 g/dl)*	11.92 (11.49-12.36)	12.26 (11.8-12.72)	0.16
CPK *(0-170 U/L)*	96.38 (75.65-117.12)	111.01 (79.44-142.59)	0.46

Note: Data of sarcopenic versus non-sarcopenic patients are presented as Mean (Confidence interval), n (%). P-values comparing the confirmed and suspected groups are obtained using the statistical test or the Mann-Whitney U statistical test, utilizing a multiple univariate analysis. Abbreviations: BMI, Body mass index; CPK, Creatine phosphokinase

### RESULTS

This analysis pertains to 140 patients who underwent the DSST and strength assessment using the Jamar hydraulic hand dynamometer (Table 1). Of these, 78 were diagnosed with sarcopenia, while 62 were not. Individuals with sarcopenia were older (mean age 76 years, range 64-94) and had lower Norton scores (*P*<0.001) compared to those without sarcopenia. Sarcopenic patients also had lower albumin levels (*P*=0.01) and higher urea levels (*P*=0.03). However, there was no significant difference in the length of hospitalization (median 2 days in both groups, *P*=0.25), creatinine levels, Hemoglobin or CPK levels (96.38 vs. 111.01 U/L, *P*=0.46). These parameters were chosen due to their potential connection to muscle function.

### Comorbidities and medications

The primary comorbidity observed in patients with sarcopenia in our cohort was atrial fibrillation and atrial flutter (p=0.012). We also observed a trend towards higher incidence of hypertension (p = 0.064). They were also more likely to be on anti-coagulation (direct oral anticoagulants, Warfarin or low molecular weight heparin) furosemide, and beta-blockers (p = 0.015, p = 0.022, and p = 0.002, respectively) see Supplementary Tables 1 and 2 in supplementary material.

To provide a broader context, we analyzed the diversity of admission reasons and the total comorbidity burden within the cohort. Admission reasons were highly variable (Supplementary Fig. 2A), and the number of diagnoses per patient did not show a significant correlation with DSS scores (Supplementary Fig. 2B-C). Additionally, we calculated an adjusted Charlson Comorbidity Index (CCI) to quantify total comorbidity burden. While CCI showed a weak univariable correlation with DSS score (Supplementary Fig. 2D), multivariable regression analyses revealed that this correlation was likely driven by collinearity with age, sex, and average hand pressure (Supplementary Fig. 2E-F).

**Table 2:** Results of DSST evaluation.

Result	Sarcopenic	Non-Sarcopenic	P.value
DSST Score	17.82 (15.95-19.69)	21.71 (19.51-23.91)	0.003
Number of symbols filled	19.46 (17.75-21.17)	23.74 (21.79-25.7)	<0.001
Percentage Correct (%)	88 (84-93)	91 (86-96)	0.587

Note: Data of sarcopenic compared to non sarcopenic patients DSST results, results shown as median (Confidence Interval). P-value is from Mann-Whitney U statistical test, utilizing a multiple univariate analysis. Abbreviations: DSST, Digit Symbol Substitution Test

### Cognitive function

Due to the inherent complexity of the DSST and the absence of a definitive normality threshold, we used the test scores primarily for comparative purposes between sarcopenic and non-sarcopenic groups (Table 2).

Individuals with sarcopenia demonstrated significantly lower DSST scores compared to non-sarcopenic patients (*P*=0.003). Sarcopenic patients also completed fewer symbols during the test (*P*<0.001).

**Figure 2.**
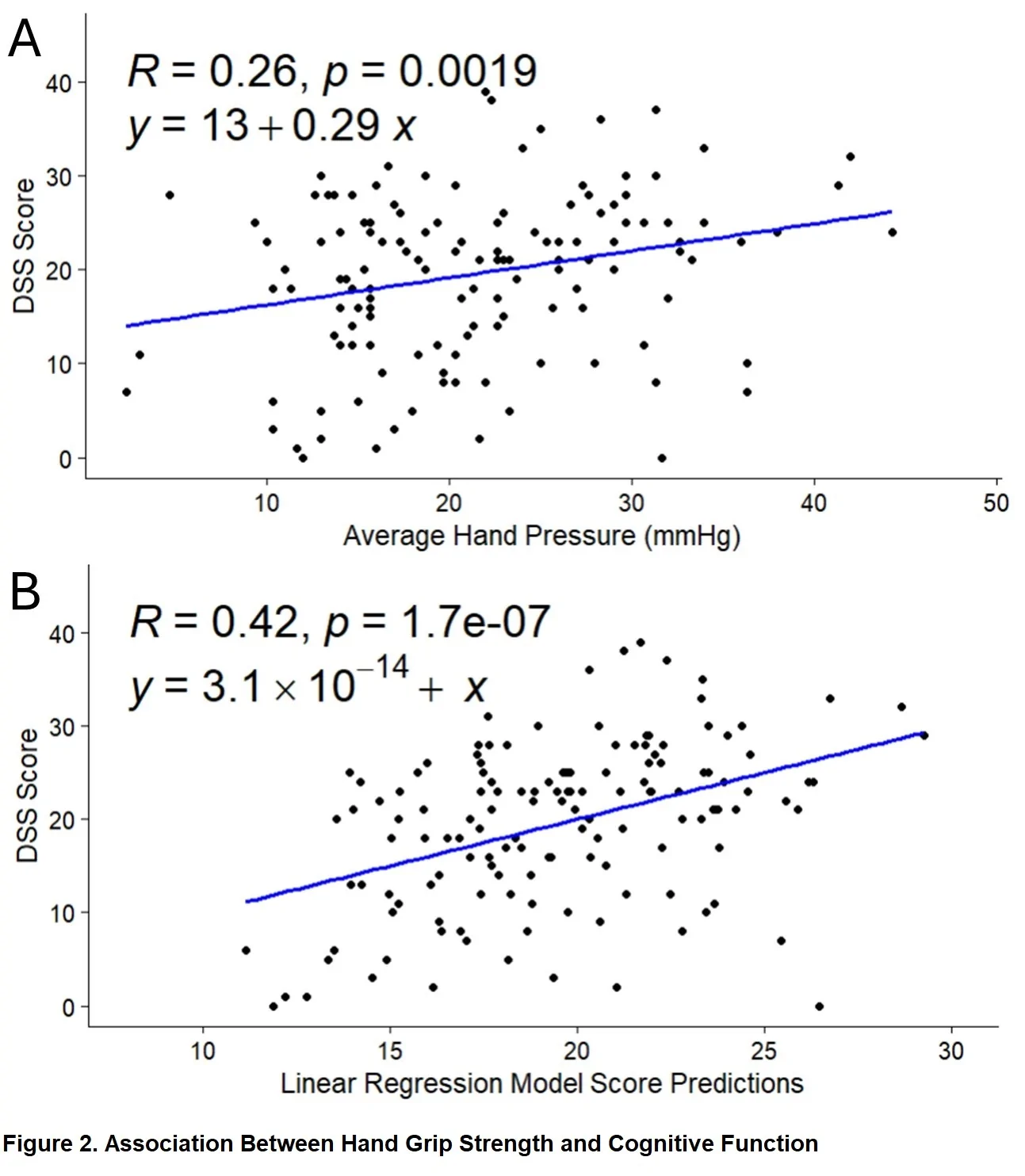
**Association Between Hand Grip Strength and Cognitive Function**. (**A**) Correlation between hand grip strength and DSST scores. (**B**) Linear regression model predicting DSST scores based on grip strength, sex, and age.

### Association between hand grip strength and cognitive function assessed by DSST

**Figure 3.**
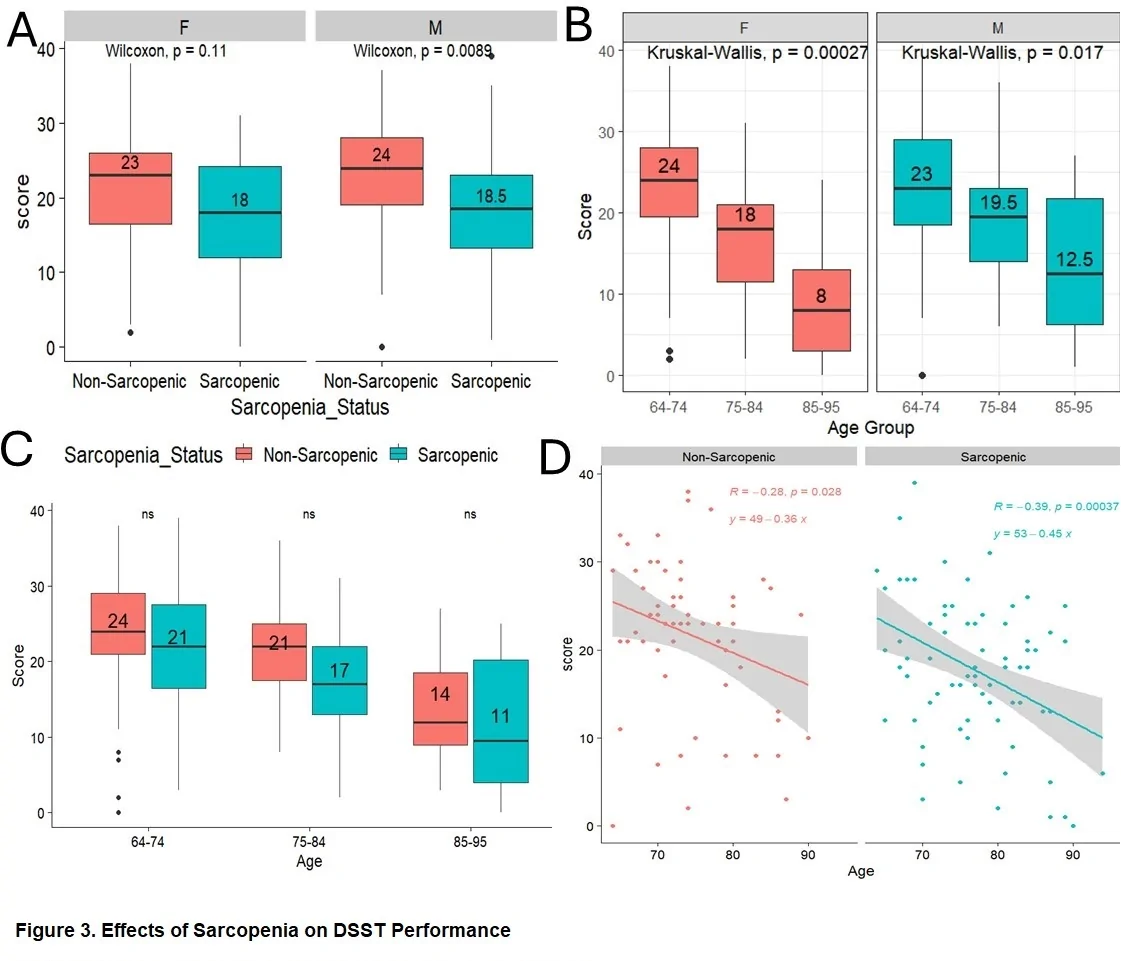
**Effects of Sarcopenia on DSST Performance**. (**A**) DSST scores stratified by sarcopenia status in males and females. (**B**) DSST scores across different age groups. (**C**) Comparison of DSST scores between sarcopenic and non-sarcopenic patients, stratified by age. (**D**) Linear regression model of DSST scores as a function of age, stratified by sarcopenia status.

A statistically significant positive correlation was found between grip strength and DSST scores (R = 0.26, p = 0.0019, β=0.3); Furthermore, a linear regression model incorporating grip strength, sex and age as predictors was used to predict DSST. The results of this analysis showed a significant relationship between the predicted DSST score and the actual scores (R = 0.42, p = 1.7e-07), suggesting that the model adequately captured variability in cognitive performance based on these variables (Fig. 2). To investigate the effects of biological sex and age on DSST performance, we conducted stratified analyses. Our findings revealed that the difference in DSST scores between sarcopenic and non-sarcopenic patients was most pronounced in males, with a significant difference (p=0.0089), while in females, the difference was not statistically significant (p=0.11). These results were independent of age, reflecting only the effects of sarcopenia. When examining age groups, both sexes demonstrated a decline in DSST scores with increasing age, as illustrated in Figure 3A-D.

We further assessed the robustness of our findings through additional regression analyses that included a broader set of potential confounders, such as comorbidity burden and medication use. These analyses confirmed the strong association between grip strength and DSST scores, with results demonstrating the model's adequacy in capturing variability in cognitive performance (see Supplementary Figure 1 for detailed regression outputs and power analysis).

## DISCUSSION

Among 140 patients hospitalized in the internal medicine ward between April 2022 to September 2023 who took part in our study, a strong correlation was found between sarcopenia assessed by upper hand grip strength and cognitive function as assessed by a digital version of the Digit Symbol Substitution Test (DSST). Individuals with sarcopenia had a significantly lower DSST score, independent of age and sex.

Utilizing linear regression analysis, we found that both age and sarcopenia’s status correlate with DSST scores. Older age was consistently associated with lower DSST scores (coefficients ranging from -0.4 to -0.48, p < 0.001), while Sarcopenic individuals also demonstrated significantly lower DSST scores (coefficient: -2.9, p = 0.044. Biological sex did not show a significant association with DSST scores.

The relationship between sarcopenia and cognitive dysfunction has been extensively explored in prior research. A systematic review demonstrated a significant association between sarcopenia and a heightened risk of cognitive impairment, irrespective of various factors such as study population and the definition of sarcopenia [18]. A study looking at hand grip strength and cognitive decline using different assessment tools, such as the Mini-Mental State Examination (MMSE), highlighted the potential of monitoring handgrip strength alongside cognitive function for insights into physical and cognitive health [29]. Longitudinal studies have further underscored the longitudinal relationship between grip strength and cognitive abilities in aging populations [30, 31].

Previous research has indicated that older adults tend to exhibit poorer performance on the DSST compared to younger counterparts, with age-related changes in brain structure and function contributing to this decline [32]. Additionally, diminished performance associated with aging may be partially attributed to a decline in motor speed [33].

Another study based on National Health and Nutrition Examination Surveys (NHANES) reported better cognitive performance in participants without sarcopenia using the DSST [34]. Notably, this study utilized survey-based data and classified sarcopenia according to appendicular lean mass (ALM). Additionally, a longitudinal study found associations between slow gait speed and a decline in DSST scores over 10 years, as well as a correlation between low handgrip strength and declines in both DSST and MMSE scores [35].

Our study is, to our best knowledge, the first to exclusively explore the relationship between sarcopenia and DSST in hospitalized patients. This unique context offers valuable insights, indicating the need for nuanced considerations when investigating the interplay between sarcopenia and cognitive outcomes.

The adoption of DSST in digital form through smartphones shows potential for efficient bedside assessments due to its ability to be integrated into routine bedside testing during admissions, akin to vital sign assessments and physical examinations. Combining it with hand-grip strength measurement may effectively identify patients with or at higher risk of sarcopenia and cognitive dysfunction.

Analyis of comorbidities and medication showed that sarcopenic patients exhibited an increased prevalence of conditions such as hypertension and atrial fibrillation, coupled with a predominance of the use of anti-coagulants, furosemide, and beta-blockers. This suggests that sarcopenic individuals may indeed manifest a unique clinical profile marked by an elevated risk of specific health conditions and a preference for certain medications. Alternatively, it raises the possibility that certain diseases confer a higher risk of developing sarcopenia.

Notably, sarcopenia's prevalence is substantial among patients with heart failure, both with preserved and reduced ejection fraction (prevalence 34-66%) according to Damluji et al [36]. Our findings align with this trend, emphasizing a connection between sarcopenia and atrial fibrillation, a link also observed in a recent study [37].

In parallel, our study corroborates the association between sarcopenia and atrial fibrillation, as well as the medications employed in its treatment, including beta-blockers, anti-coagulants, and diuretics. However, unraveling the causal direction of this association remains a complex endeavor. Determining whether sarcopenia acts as a causative factor, or a consequence of these conditions is pivotal. The intricate relationship hints at the possibility of a bidirectional association, warranting careful consideration in future research endeavors.

Our study has inherent limitations that warrant consideration when interpreting the findings. The relatively modest sample size may restrict the generalizability of our results. Additionally, the non-randomized nature of participant recruitment and the exclusion of individuals with visual impairments from the DSST evaluation introduce potential selection bias. Attempts to address the limitations posed by small smartphone screens, particularly for patients aged 65 or older unfamiliar with such technologies, using larger tablet screens were hindered by technical issues and network problems. Another limitation of the current study is its cross-sectional design, which precludes causal inferences between sarcopenia and cognitive decline. Although our findings suggest a strong association, the temporal direction of this relationship remains unclear. Longitudinal studies are needed to determine whether sarcopenia precedes cognitive decline or vice versa, as well as to explore potential shared underlying mechanisms, such as chronic inflammation or reduced physical activity. Such studies would also allow for the investigation of whether interventions targeting sarcopenia could mitigate cognitive decline over time.

Understanding this bidirectional relationship has practical implications for hospital assessment protocols. Integrating assessments for both sarcopenia and cognitive function in clinical settings can aid in early identification and intervention. This holistic approach may help to prevent the progression of both conditions and improve patient outcomes.

Furthermore, while the DSST is a widely used cognitive assessment tool, its sensitivity to specific cognitive domains may limit its ability to comprehensively capture cognitive function. Exploring alternative assessments or employing a combination of tools could enhance our understanding. To address potential usability challenges of digital tools like smartphones for administering the DSST, we implemented a user-centered approach. All participants underwent a brief orientation session to familiarize themselves with the device and test interface. The digital version was designed with a simplified interface, larger font sizes, and clear instructions to enhance accessibility for older adults. Additionally, trained research staff were present to provide real-time assistance if participants encountered difficulties. These measures ensured that test performance accurately reflected cognitive abilities rather than digital proficiency.

Acknowledging these limitations is essential for a nuanced interpretation of our study's findings and for guiding future research endeavors aimed at unraveling the complexities of sarcopenia, cognitive function, and associated clinical parameters across diverse patient populations.

Our findings add to the growing body of evidence suggesting an association between sarcopenia and cognitive dysfunction in older adults. The precise mechanisms underlying this association remain elusive, but several potential pathways warrant further investigation.

Routine assessment of sarcopenia through handgrip strength and cognitive function via the DSST offers valuable insights into early indicators of adverse clinical outcomes, such as prolonged hospital stays, increased readmission rates, and functional decline. Early identification allows for the implementation of targeted interventions, including physical rehabilitation, nutritional optimization, and cognitive training, which could enhance functional independence and reduce healthcare burdens. Future research should explore the efficacy of these interventions to strengthen the evidence for routine assessments in improving patient outcomes.

In conclusion, our study offers a practical tool of initial effective screening of sarcopenia and cognitive function, as well as their correlation in hospitalized patients. The study underscores the importance of integrating sarcopenia and cognitive assessments into routine clinical care for older adults. Practical screening tools, such as handgrip dynamometers and the DSST, can identify high-risk patients and guide targeted interventions. Further research with larger cohorts is warranted to elucidate the mechanisms linking sarcopenia, cognitive function, and overall health outcomes, allowing for accessible targeted interventions and personalized care strategies in hospitalized older adults.

## Supplementary Materials

The Supplementary data can be found online at: www.aginganddisease.org/EN/10.14336/AD.2024.1676.

## Data Availability

The datasets used and or analysed during the current study are available from the corresponding author on reasonable request
