# Supplementary material for: Sarcopenia and Cognitive Decline in Hospitalized Older Adults from a Prospective Study
Source: Aging Dis. 2025 Feb 25;17(1):578–87. doi: 10.14336/AD.2024.1676 (PMC12727076; doi:10.14336/AD.2024.1676)
Supplement: Supplementary file 1 — The Supplementary data can be found online at: www.aginganddisease.org/EN/10.14336/AD.2024.1676. [file AD-17-1-578-s.pdf]

SUPPLEMENTARY DATA

Sarcopenia and Cognitive Decline in Hospitalized Older Adults from a Prospective Study Sarcopenia and Cognition in Hospitalized Adults

Sapir Kon-Kfir, Tali Cukierman-Yaffe, Haim Krupkin, Ana Belkin, Gadi Shlomai, Jonathan Bleier, Shiri Weinstein, Liora Bruckmayer, Elad Prinz, Alon Kaplan, Michal Goldenberg Shraga, Dana Lev, Shahar Dekel, Noa Shalmon, Nurit Tsarfaty, Niv Reiss, Evelyne Bischof, Avshalom Leibowitz

© 2025. Kon-Kfir S. et al. Published online at http://www.aginganddisease.org/EN/10.14336/AD.2024.1676

SUPPLEMENTARY DATA

Supplementary Table 1. Comorbidities in the study cohort of sarcopenic and non-sarcopenic patients.

Comorbidity	Sarcopenic with Comorbidity (%)	Not-Sarcopenic with Comorbidity (%)	P. value
Atrial Fibrillation/Flutter	19.3	6.4	**0.012**
Hypertension	44.6	28.1	0.064
Cancer	15	5.7	0.068
Chronic Heart Failure	21.6	11.5	0.18
Diabetes Mellitus	22.9	16.4	0.765
COPD	7.9	5.7	1
Previous CVA/TIA	5.8	6.6	0.627

Abbreviations: COPD, Chronic Obstructive Pulmonary Disease; CVA, Cerebrovascular accident; TIA, transient ischemic attack

Supplementary Table 2. Medications in the study cohort of sarcopenic and non-sarcopenic patients.

Medication	Sarcopenic Treated (%)	Not-Sarcopenic Treated (%)	P. value
Beta Blockers	37.1	17.1	**0.002**
Anti Coagulation	20	7.1	0.015
Furosemide	12.1	2.9	0.022
ACEi/ARB	34.5	23	0.272
Benzodiazepines	5.7	7.1	0.437
SSRI	7.1	8.6	0.411
SNRI	0.7	2.1	0.457
CCB	17.1	15.7	0.683
Alpha Blockers	12.2	10.1	1
Aspirin	24.3	21.4	0.693
Dual Antiplatelet Therapy	4.3	3.6	1
SGLT2 Inhibitor	5	4.3	1
GLP1 Receptor Agonist	3.6	3.6	0.979
Metformin	10.7	10.7	0.615

Abbreviations: ACEi, Angiotensin-converting-enzyme inhibitors; ARB, Angiotensin receptor blockers; SSRI, Selective serotonin reuptake inhibitors; SNRI, Serotonin and norepinephrine reuptake inhibitors; CCB, Calcium channel blockers; DAPT, Dual Antiplatelet Therapy; SGLT2, Sodium-glucose cotransporter-2 ;GLP1, Glucagon-like peptide-1.

© 2025. Kon-Kfir S. et al. Published online at http://www.aginganddisease.org/EN/10.14336/AD.2024.1676

SUPPLEMENTARY DATA

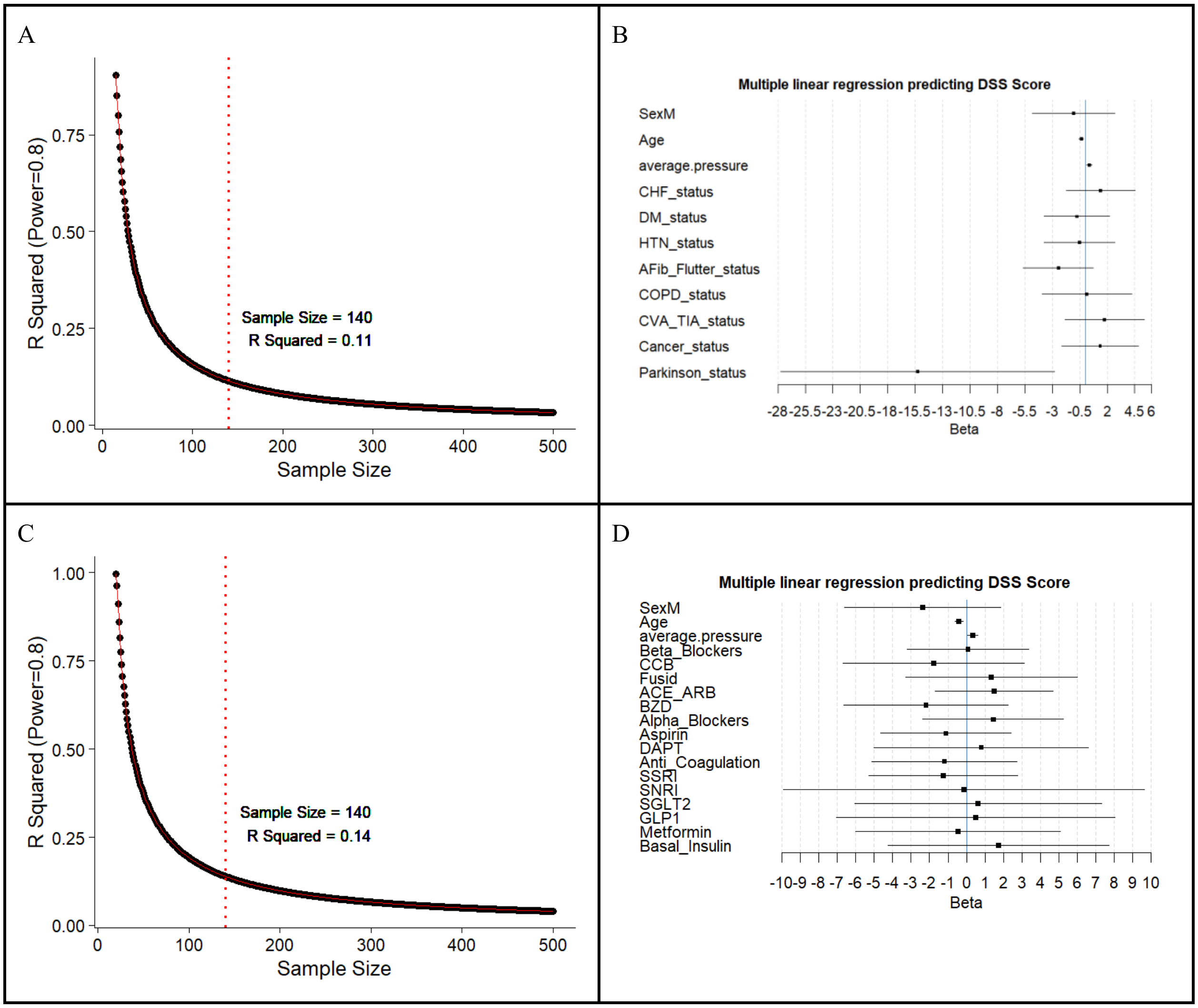

Figure S1. Power Analysis and Results of Multiple Linear Regression Models Assessing Predictors of DSS Score. A) Simulated sample size requirements for multiple regression models with 12 predictors, a significance threshold of <0.05, and power = 0.8, as a function of R-squared thresholds. **B)** Results of a multiple regression analysis predicting DSS score based on sex, age, average hand pressure, and disease statuses, including chronic heart failure (CHF_status), diabetes mellitus (DM_status), hypertension (HTN_status), atrial fibrillation/flutter (AFib_Flutter_status), chronic obstructive pulmonary disease (COPD_status), prior cerebrovascular accident or transient ischemic attack (CVA_TIA_status), cancer (Cancer_status), and Parkinson's disease (Parkinson_status). Model power = 0.9965, Adjusted R-squared = 0.166, overall model p-value < 0.001. **C)** Simulated sample size requirements for multiple regression models with 18 predictors, a significance threshold of <0.05, and power = 0.8, as a function of R-squared thresholds. **D)** Results of a multiple regression analysis predicting DSS score based on sex, age, average hand pressure, and medication use, including beta blockers (Beta_Blockers), calcium channel blockers (CCB), fusidic acid (Fusid), angiotensin-converting enzyme inhibitors or angiotensin II receptor blockers (ACE_ARB), benzodiazepines (BZD), alpha blockers (Alpha_Blockers), aspirin (Aspirin), dual antiplatelet therapy (DAPT), anticoagulation therapy (Anti_Coagulation), selective serotonin reuptake inhibitors (SSRI), serotonin-norepinephrine reuptake inhibitors (SNRI), sodium-glucose cotransporter 2 inhibitors (SGLT2), glucagon-like peptide-1 receptor agonists (GLP1), metformin (Metformin), and basal insulin (Basal_Insulin). Model power = 0.979, Adjusted R-squared = 0.099, overall model p-value = 0.027.

© 2025. Kon-Kfir S. et al. Published online at http://www.aginganddisease.org/EN/10.14336/AD.2024.1676

SUPPLEMENTARY DATA

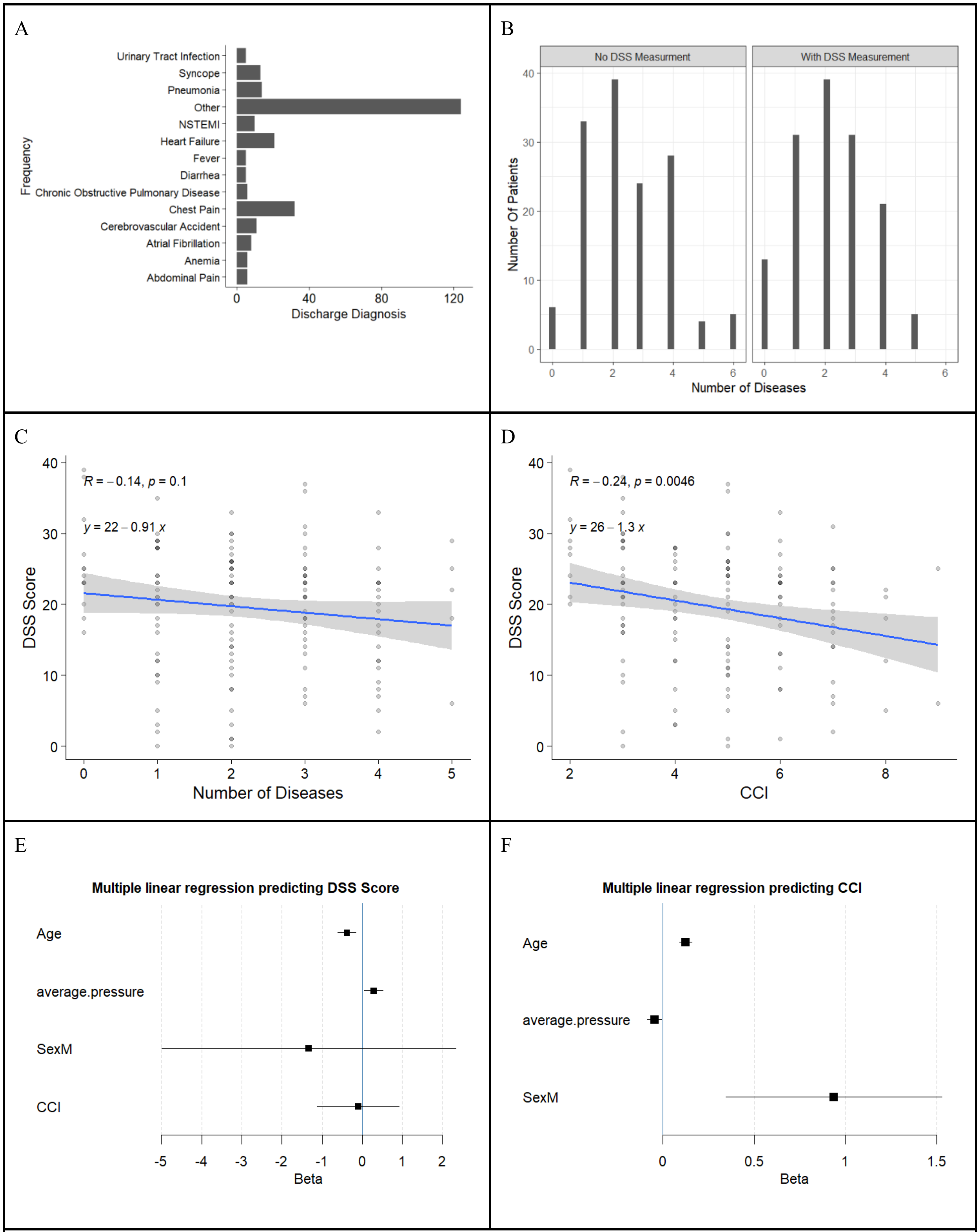

Supplementary Figure 2. Analyses of Admission Reasons, Comorbidity Burden, and Their Association with DSS Scores. A) Distribution of primary admission reasons among the cohort. Admission reasons occurring in fewer than five patients were excluded to maintain patient anonymity. **B)** Number of diagnoses per patient in the cohort. **C)** Correlation between the number of diagnoses and DSS score. The analysis revealed no significant relationship

© 2025. Kon-Kfir S. et al. Published online at http://www.aginganddisease.org/EN/10.14336/AD.2024.1676

between these variables. **D)** Univariable analysis of the association between the adjusted Charlson Comorbidity Index (CCI) and DSS score. A weak correlation was observed. **E)** Multivariable regression analysis of DSS score as a function of CCI, age, sex, and average hand pressure. This analysis showed no significant independent association between CCI and DSS score. **F)** Multivariable regression analysis of CCI as a function of age, sex, and average hand pressure. Significant associations were observed for each of these variables, indicating that the univariable correlation between CCI and DSS score is likely driven by collinearity.

© 2025. Kon-Kfir S. et al. Published online at http://www.aginganddisease.org/EN/10.14336/AD.2024.1676
